# Supplementary material for: Development and Validation of an Auricular Acupuncture Protocol for the Management of Chemotherapy-Induced Nausea and Vomiting in Cancer Patients
Source: Healthcare (Basel). 2024 Jan 16;12(2):218. doi: 10.3390/healthcare12020218 (PMC10815809; doi:10.3390/healthcare12020218)
Supplement: Supplementary file 1 [file healthcare-12-00218-s001.zip › healthcare-2798123-supplementary.pdf]

Supplementary Material

Table S1 - Study procedures and sequence of assessments and interventions.

Weeks	1	2	3	4	5	6	7
**Initial assessment** (sociodemographic and clinical characterization questionnaire; visual numeric scale; Morrow Assessment of Nausea and Emesis)	X						
**AA session 1**	X						
**AA session 2**		X					
**AA session 3**			X				
**AA session 4**				X			
**AA session 5**					X		
**Final evaluation** (Morrow Assessment of Nausea and Emesis)						X	
**Follow-up evaluation** (Morrow Assessment of Nausea and Emesis)							X

Table S2 - Final version of the auricular acupuncture protocol for chemotherapy-induced nausea and vomiting.

Item description	Final version of the proposed protocol
**Acupuncture style**	Auricular acupuncture (AA) based on the precepts of Traditional Chinese Medicine (TCM)
**Reasoning for treatment provided, based on**	AA is a specialized approach to TCM/acupuncture, which uses the auricular microsystem for the treatment, prevention, and diagnosis of health conditions [36]. It is currently used in more than 249 areas of the health sciences, including oncology [36]. The AA physiological

**historical context, literature sources, and/or consensus methods, with references where suitable**	mechanism of action is still under study [14]. However, it is believed to be neurologically based on stimulation of the trigeminal and vagus nerves, thus generating neuromodulation in the Central Nervous System (CNS). In addition, the stimulation of auricular points may be related to an increase in vagal tone, as well as regulations in the gastrointestinal, endocrine, cardiovascular, and respiratory systems [14,15].
**Extent to which treatment may vary**	All patients will receive the same treatment protocol in all five sessions. The final version of the protocol will be based on the results of a previous systematic review [17], on content validation by specialists in the field, and on clinical validation.
**Number of device insertions per subject and per session**	Seven auricular seeds will be applied to each subject per session.
**Names (or location if no standard name) of points used (uni/bilateral)**	Shénmén (TF4) shenmen, Jiaogan (AH6a) sympathetic nerve, Wèi (CO4) stomach, Pí (CO13) spleen, Gän (CO12) liver, Pízhìxià (AT4) subcortex, and bënmén (C03) cardia. These points will be applied unilaterally, alternating ears at each session.
**Depth of insertion**	Not applicable.
**Response sought**	Patients will be instructed to press the seeds at three different times: in the morning (3 times), in the afternoon (3 times), and in the evening (3 times), and whenever they feel nauseous or vomit, for approximately 30 seconds at each point, until the ear becomes slightly hyperemic [39], or until they feel slight discomfort or pain [29,37]- with a sensation of "*deqi*".
**Device stimulation**	Manual seed acupressure.
**Device retention time**	Patients will be instructed to keep the seeds fixed in their ears for a period of seven days.<br><br>In the event that they are removed before this period (intentionally or unintentionally), patients will be instructed to inform the researcher.
**Device type**	Vaccaria seeds
**Number of treatment sessions**	Five sessions
**Frequency and duration of treatment sessions**	Once a week, for approximately 20 minutes each session. The entire treatment will last approximately five weeks.
**Details of other interventions administered**	No other interventions will be applied.
**Setting and context of treatment, including instructions to practitioners, and**	Only a qualified interventionist will apply the intervention. After attaching the seeds, participants will be instructed to press each point for 30 seconds, three times a day: in the morning, afternoon, and evening, and whenever they feel

**information and explanations to patients**	nauseous or vomit [38]. In addition, they will be instructed to keep the devices inserted until the next AA session, for a period of seven days, or to remove them in the event of discomfort, allergic processes and/or itching [40]. They will also be instructed on how to perform hygiene and how to maintain the seeds in the ear, as well as the possible discomfort resulting from the procedure. These guidelines will be reinforced with each new session. To reinforce the necessary care, patients will receive a leaflet containing guidelines on how to maintain the stitches, the scheduled days for the AA sessions and evaluations, as well as a contact number of the researcher in charge.
**Description of participating acupuncturists**	Academic qualifications: Nurse. Specialization in classical systemic acupuncture, with 1200 hours / Minimum of two years of experience; 80-hour AA course /Minimum of four years of experience.
